# Taurine attenuates arsenic-induced pyroptosis and nonalcoholic steatohepatitis by inhibiting the autophagic-inflammasomal pathway

**DOI:** 10.1038/s41419-018-1004-0

**Published:** 2018-09-20

**Authors:** Tianming Qiu, Pei Pei, Xiaofeng Yao, Liping Jiang, Sen Wei, Zhidong Wang, Jie Bai, Guang Yang, Ni Gao, Lei Yang, Shuangyue Qi, Rushan Yan, Xiaofang Liu, Xiance Sun

**Affiliations:** 1 Department of Occupational & Environmental Health, School of Public Health, Dalian Medical University, Dalian, China; 2 Department of Nutrition & Food Safety, School of Public Health, Dalian Medical University, Dalian, China; 3 Global Health Center, Dalian Medical University, Dalian, China

## Abstract

Arsenic exposure causes nonalcoholic steatohepatitis (NASH). Inflammation is a key contributor to the pathology of nonalcoholic fatty liver disease (NAFLD), including NASH. However, it is unclear how arsenic induces inflammation. In mouse livers, we show that arsenic trioxide (As_2_O_3_) induced NASH, increased autophagy and NLRP3 inflammasome activation, increased lipid accumulation, and resulted in dysregulation of lipid-related genes. Supplemented with taurine (Tau) attenuated the inflammation and autophagy caused by As_2_O_3_. In HepG2 cells, we found that As_2_O_3_-induced pyroptotic cell death was dependent upon the activation of NLRP3 inflammasome, which was CTSB-dependent. In addition, inhibiting autophagy alleviated the As_2_O_3_-induced increase of cytosolic CTSB expression and subsequent release of LDH, activation of the NLRP3 inflammasome, and pyroptosis. Moreover, we found that Tau alleviated As_2_O_3_-induced elevation of autophagy, CTSB expression, and activation of the NLRP3 inflammasome, and reduced the release of LDH, pyroptotic cell death, and inflammation. Interestingly, As_2_O_3_-induced lipid accumulation could not be alleviated by either inhibition of autophagy nor by inhibition of CTSB. Additionally, neither inhibition of the NLRP3 inflammasome or Tau treatment could alleviate lipid accumulation. These results demonstrated that As_2_O_3_-induced pyroptosis involves autophagy, CTSB, and the NLRP3 inflammasome cascade, and that Tau alleviates As_2_O_3_-induced liver inflammation by inhibiting the autophagic-CTSB-NLRP3 inflammasomal pathway rather than decreasing lipid accumulation. These findings give insight into the association of autophagy, inflammation, pyroptosis, and NASH induced by As_2_O_3_.

## Introduction

Many countries face groundwater quality problems of enormous proportions from both industrial and natural sources. Populations at risk of exposure to excessive levels of arsenic, a groundwater contaminant, have been emerging since the 1960s^1^. Arsenic exposure initiates a multitude of biological alterations which cause liver cirrhosis, pancreatic damage, and cancers of the skin, liver, and kidney^2,3^. The liver, a major site of arsenic metabolism, is a known target of arsenic toxicity^4^. Arsenic-induced liver injury can progress to fibrosis, cirrhosis, and even cancer^5^.

NASH constitutes a major threat to global health, and increasing studies are focusing on arsenic-induced non-alcoholic fatty liver disease (NAFLD) and nonalcoholic steatohepatitis (NASH)^6–9^. However, the molecular mechanism by which arsenic causes NASH/NAFLD is unclear. NAFLD is the main manifestation of arsenic-induced liver injury, usually accompanied by obesity, dyslipidemia, and insulin resistance^10^. NAFLD is a major contributor to liver-related illness and death^11^. NAFLD prevalence in China is 15–30%^12^, which is similar to that of Europe (20–30%)^13^, but high compared to other major Asian countries^14^. The NAFLD disease spectrum is broad, starting with lipid accumulation in the liver^15^, and about 30–40% of NAFLD patients will develop NASH^16^. However, it is unclear how NAFLD progresses to NASH after arsenicosis.

In recent years, studies have suggested that the inflammatory response is a central link in upstream insulin resistance and steatosis, and that downstream cell damage plays a vital role in the progression of NAFLD to NASH^17^. Pattern recognition receptors (PRRs) are also involved in the progression of NAFLD^18^. Inflammasomes, composed of a cytosolic sensor, the adaptor apoptosis-associated speck-like protein containing a CARD (ASC) and the effector pro-caspase-1, are intracellular multiprotein complexes which play important roles in innate immune defenses by activating caspase-1^19,20^. Nucleotide oligomerization domain-like receptor proteins (NLRPs), particularly NLRP3, acting as novel PRRs, interact with ASC and pro-caspase-1 to activate caspase-1. This leads to maturation and secretion of interleukin (IL)-1β and IL-18^19^, and eventually to pyroptosis^21^.

Pyroptosis, a form of inflammatory cell death, is different from apoptosis in that it is uniquely dependent on caspase-1. Pyroptosis is accompanied by plasma membrane rupture, cytoplasmic swelling, osmotic lysis, DNA cleavage, NLRP3 inflammasome activation, and the release of pro-inflammatory cellular contents^22,23^. In addition to a priming signal which activates NF-κB through binding of the toll-like receptor (TLR), lipopolysaccharide (LPS) to upregulate NLRP3 and pro-IL-1b are also essential. Increased mRNA levels of NLRP3 in inflammatory cells were found in human NAFLD and type 2 diabetes mellitus^24^. Additionally, several environmental crystallines and particulate irritants have been shown to activate NLRP3 inflammasomes, and inflammasome activation has been linked to silicosis and asbestosis^25–27^. Recently, it was reported that cadmium selenide (CdSe)/zinc sulfide (ZnS) quantum dots induce hepatocyte pyroptosis and liver inflammation via NLRP3 inflammasome activation^28^. However, it is unknown whether arsenic activates NLRP3 inflammasomes, and it is inconclusive as to whether the activation of NLRP3 inflammasomes is involved in arsenicosis.

To date, the unequivocal upstream activation mechanism of NLRP3 inflammasomes has not yet been determined. However, it has been reported that cathepsin B (CTSB) mediates the activation of the NLRP3 inflammasome. Increased cytoplasm levels of CTSB contribute to NLRP3 inflammasome activation, and CTSB suppression with a chemical inhibitor decreased the activation of the NLRP3 inflammasomes^29,30^. Moreover, the level of CTSB in the cytoplasm was associated with autophagic flux. The degree of autophagy was elevated, and degradation of autophagolysosomes resulted in the cytosolic release of CTSB, which is linked to activation of the NLRP3 inflammasome^26,31,32^. Nevertheless, whether arsenic-induced autophagy, degradation of autophagolysosome, or increase of cytoplasm CTSB lead to hepatocyte inflammation and pyroptosis have not been investigated.

Taurine (Tau), a sulfur-containing β-amino acid, is a major free intracellular amino acid present in many tissues of humans and animals^33^. The recognized metabolic function of taurine in the liver is its conjugation with bile acids, which is important for bile secretion and lipid digestion^34,35^. In addition, antioxidative and anti-inflammatory effects may be important for the prevention of NASH by taurine^36^. However, the exact target of taurine during this process and whether taurine plays the same role in arsenic-induced NASH are unclear.

In our study, we investigated the association between arsenic-induced autophagy, the cytosolic release of lysosomal contents (CTSB), activation of NLRP3 inflammasome and caspase-1-dependent pyroptosis, and NASH.

## Results

### Effects of taurine against As_2_O_3_-caused NASH in mice livers

**Fig. 1:**
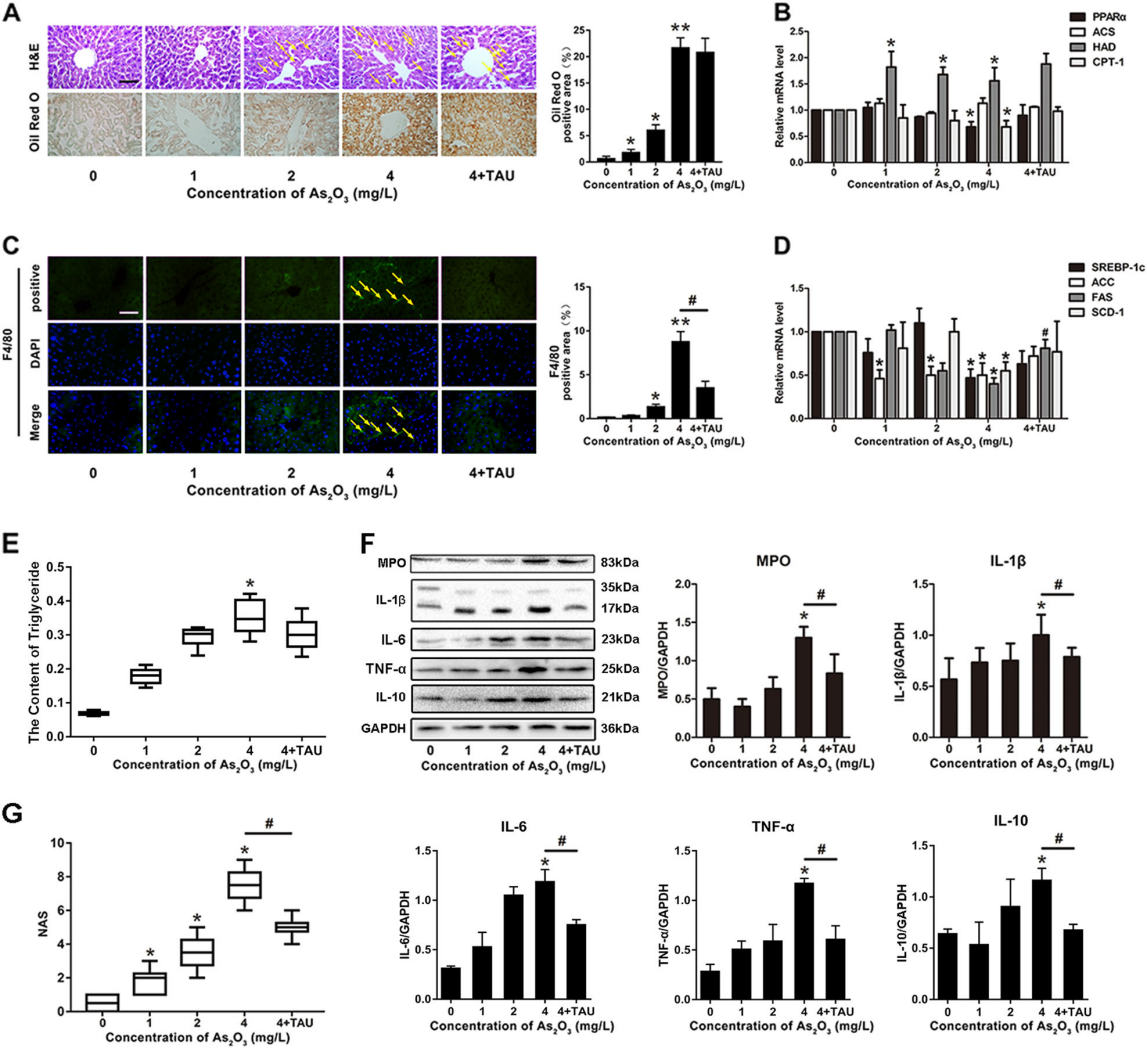
As_2_O_3_ induced inflammation and accumulation of lipids in mouse livers. **a** HE and Oil Red O staining of liver sections after As_2_O_3_ administration and pretreatment with Tau. Yellow arrows indicate steatosis. **b**–**d** mRNA levels of lipid-associated genes in liver tissues after exposure to As_2_O_3_ and pretreatment with Tau, performed by qPCR. **c** F4/80 immunofluorescence staining of liver sections following As_2_O_3_ and Tau treatment. Yellow arrows indicate positive staining. **e** The content of triglyceride in mice livers. **f** The expression of liver inflammation-related proteins following exposure to As_2_O_3_ and Tau. The protein fraction was analyzed by western blot. **g** NAS in mouse livers. Values are mean ± SD, and *n* = 6. *P < 0.05 compared with the control group, ^#^*P* < 0.05 compared with the 4 mg/L group; Scale bar = 100 μm

The toxicology of As_2_O_3_ was measured by H&E staining, Oil Red O staining, western blot, and immunofluorescence (IF) (Fig. 1). Compared with control group, steatosis and lipid accumulation were increased in the 4 mg/L group as shown by HE and Oil Red O staining (Fig. 1a). Furthermore, to measure the effect of As_2_O_3_ on liver lipids, we performed real time-qPCR. Lipid-associated genes were dysregulated in 4 mg/L group mice compared with control group (Fig. 1b–d). Similar change have also been observed in the detection of triglyceride content (Fig. 1e). Compared with the control group, As_2_O_3_-induced inflammation in liver tissue was increased, which was further confirmed by F4/80 IF staining (Fig. 1c), and MPO, IL-1β, IL-6, IL-10 and TNF-α immunoblotting (Fig. 1f). However, after supplemented with Tau, the level of inflammation was decreased, but the steatosis, accumulation of lipid, dysregulation of lipid genes and increased triglyceride content were not attenuated (Fig. 1a–f). NAS of the mouse livers (Fig. 1g) showed that the control and 1 mg/L group did not exhibit signs of NASH, the 2 mg/L group had scores that were borderline, and the 4 mg/L group had definitive NASH. The 4 mg/L group had significantly higher NAS than the control animals, and the TAU group had significantly lower NAS than 4 mg/L group. This decrease was mainly due to the score of lobular inflammation and ballooning rather than the score of steatosis.

### Taurine inhibits As_2_O_3_-induced upregulation of autophagy, activation of NLRP3 inflammasome, and pyroptosis in mouse livers

**Fig. 2:**
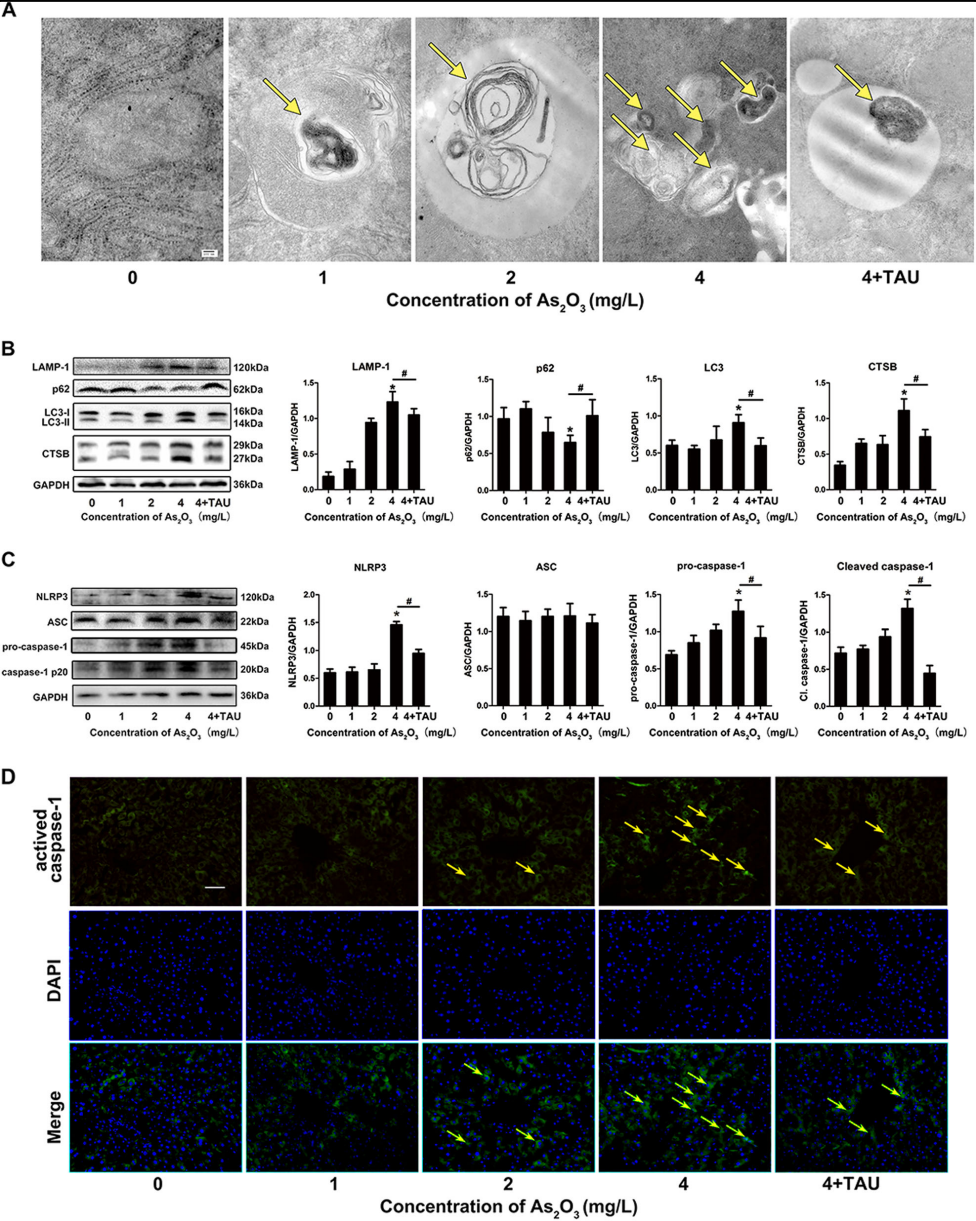
As_2_O_3_ induced autophagy, activation of NLRP3 inflammasome, and activation of caspase-1 in mouse livers. **a** Ultrastructural features of As_2_O_3_-treated mouse livers by transmission electron microscopy. Yellow arrows indicate autophagosomes (scale bar = 100 nm). **b** The protein level and densitometric analyses of LAMP-1, p62, LC3, and CTSB in mouse liver tissues. **c** NLRP3, ASC, and caspase-1 expression and densitometric analyses in mouse liver tissues. **d** Activated caspase-1 detected by IF. Yellow arrows indicate positive staining. Values are mean ± SD, and *n* = 6. *P < 0.05 compared with the control group, ^#^*P* < 0.05 compared with the 4 mg/L group; Scale bar = 200 μm

We utilized transmission electron microscopy and western blot to observe the ultrastructure and level of intracellular autophagy in As_2_O_3_-treated mice to investigate whether autophagy was involved in As_2_O_3_-induced toxicity. The autophagosomes and autophagolysosomes were observed in the mouse livers (Fig. 2a). The levels of LC3-II and LAMP-1 were elevated, p62 levels were decreased, and cytoplasmic CTSB levels increased simultaneously with increasing concentration of As_2_O_3_ (Fig. 2b). Additionally, NLRP3 and caspase-1 p20 expression increased in hepatocytes with increasing concentrations of As_2_O_3_ (Fig. 2c). Given the activation of NLRP3 inflammasome after As_2_O_3_ treatment, we examined whether As_2_O_3_ induced pyroptosis in hepatocytes. As previously mentioned, pyroptosis depends on caspase-1 activation. IF was performed to measure activated caspase-1 in hepatocytes. Hepatocytes treated with As_2_O_3_ displayed an increase in positive staining of activated caspase-1 (Fig. 2d). Moreover, all the above results were attenuated by supplemented with Tau (Fig. 2a–d).

### As_2_O_3_-induced hepatocyte pyroptosis depends on activation of the NLRP3 inflammasome

**Fig. 3:**
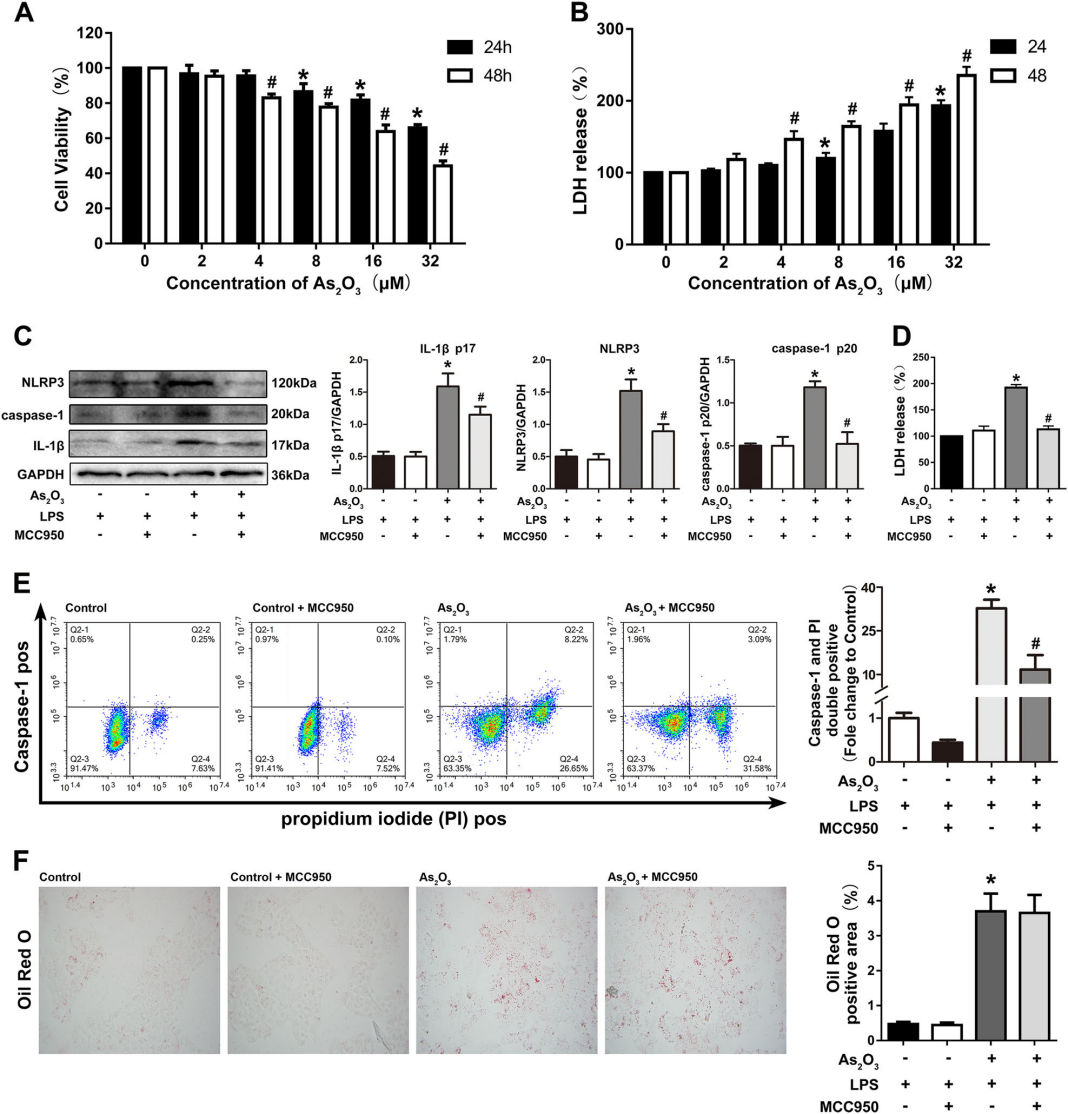
Pyroptosis induced by As_2_O_3_ in hepatocytes is NLRP3 inflammasome-dependent. **a** Effects of As_2_O_3_ on cell viability in HepG2 cells. **b** The release of LDH after treatment with As_2_O_3_. (**c**)The efficiency of MCC950, and its effect on caspase-1 activation and IL-1β production in As_2_O_3_-treated HepG2 cells. **d** Effects of As_2_O_3_ and MCC950 on LDH release rate in HepG2 cells. **e** Effects of MCC950 on As_2_O_3_-induced double-positivity for PI and activated caspase-1 staining. **f** Effects of MCC950 on the As_2_O_3_-induced accumulation of lipids. **a** *P < 0.05 compared with 24 h control, #*P* < 0.05 compared with 48 h control. **b**–**i** Values are mean ± SD, and n = 3. *P < 0.05 compared with control group, #*P* < 0.05 compared with the As_2_O_3_ group; Scale bar = 50 μm

We inhibited the expression of NLRP3 using MCC950, a NLRP3-specific inhibitor, to assess the role of the NLRP3 inflammasome in As_2_O_3_-induced pyroptosis in hepatocytes. The HepG2 cells were pretreated with MCC950 (5 μM) and LPS (1 μg/ml) prior to treatment with 4 μM As_2_O_3_ for 48 h. Before that, the effect of As2O3 on the viability of HepG2 cells was detected (Fig. 3a, b). As shown, the protein levels of NLRP3, caspase-1 p20, and IL-1β were increased by As_2_O_3_ treatment, indicating that NLRP3 inflammasome activation was increased (Fig. 3c). MCC950 diminished caspase-1 p20 expression and IL-1β production in HepG2 cells (Fig. 3c). Additionally, MCC950 inhibited the release of LDH caused by As_2_O_3_ (Fig. 3d). In addition, flow cytometry (FCM) was performed to examine caspase-1 activation and membrane pore formation in HepG2 cells. MCC950 decreased the amount of pyroptotic cell death induced by As_2_O_3_ treatment, as evidenced by decreased caspase-1 and PI double-positivity (Fig. 3e). However, the As_2_O_3_-induced accumulation of lipid was not affected by MCC950, as shown by Oil Red O staining (Fig. 3f).

### As_2_O_3_-induced NLRP3 inflammasome activation and pyroptosis are mediated by cytoplasmic CTSB

**Fig. 4:**
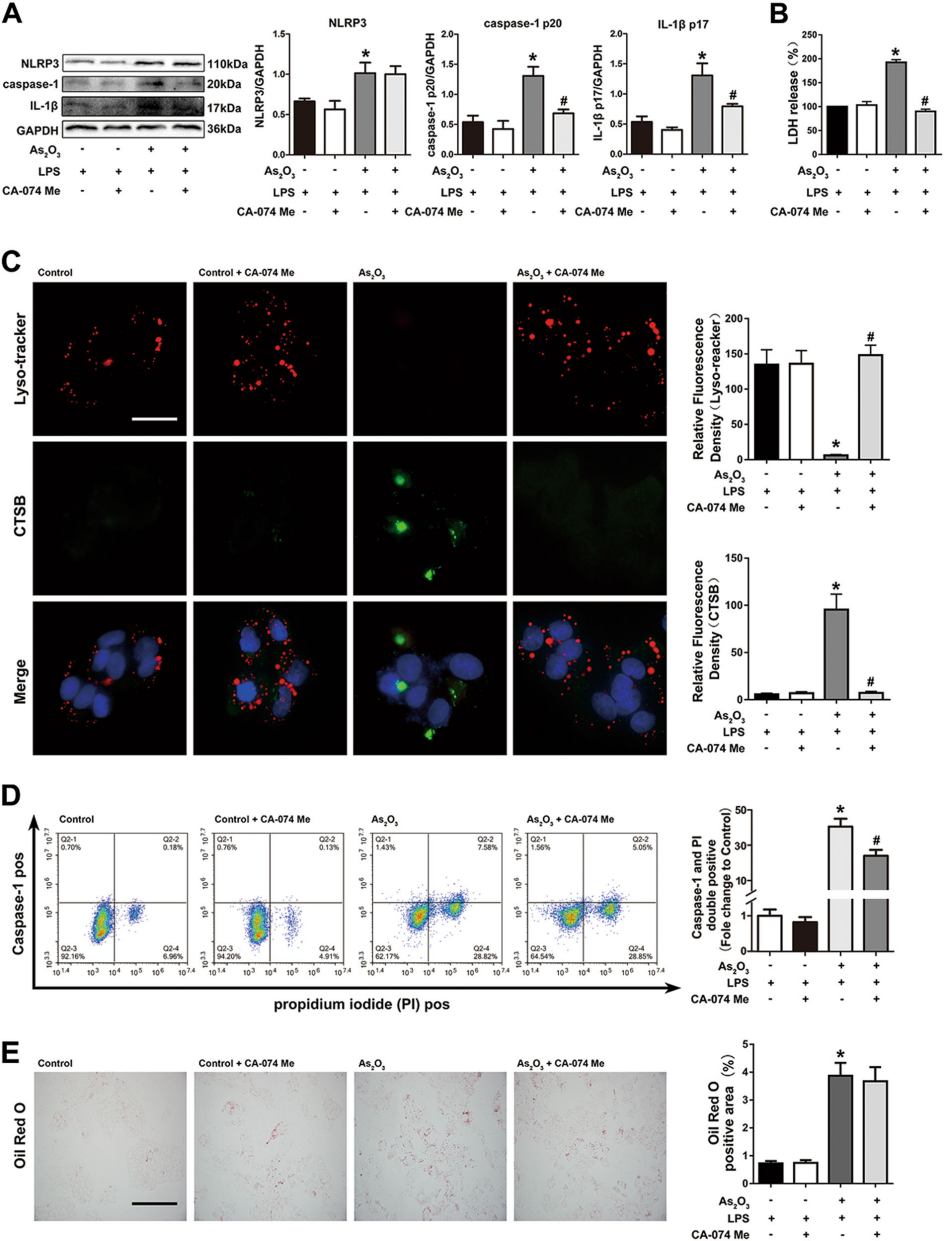
CTSB mediated the As2O_3_-induced activation of NLRP3 inflammasomes and pyroptotic cell death. **a** The efficiency of CA-074 Me, and its effect on caspase-1 activation and IL-1β production in As_2_O_3_-treated HepG2 cells, and densitometric analyses of NLRP3, caspase-1 p20, and IL-1β. **b** Effects of As_2_O_3_ and CA-074 on LDH release rate in HepG2 cells. **c** Effects of CA-074 Me on As_2_O_3_-induced double-positive PI and activated caspase-1 staining. **d** Detection of cytosolic and lysosomal CTSB levels after As_2_O_3_ and CA-074 Me treatments. **e** Effects of CA-074 Me on the As_2_O_3_-induced accumulation of lipid. Values are mean ± SD, and n = 3. *P < 0.05 compared with the control group, #*P* < 0.05 compared with the As_2_O_3_ group; Scale bar = 50 μm

CTSB has been shown to be one mechanism by which NLRP3 inflammasomes can be activated, and increased levels of CTSB were observed in mouse livers. Thus, we pretreated cells with CA-074 Me, an inhibitor of CTSB, to measure the role of CTSB in As_2_O_3_-induced activation of the NLRP3 inflammasome. The increased expression of caspase-1 p20, IL-1β, and release of LDH induced by As_2_O_3_ were downregulated by CA-074 Me (Fig. 4a, b). As observed by microscopy, CA-074 Me reduced cytosolic CTSB levels (Fig. 4c). Moreover, the amount of pyroptotic cell death induced by As_2_O_3_ treatment was also decreased in the presence of the CTSB inhibitor (Fig. 4d). Similar to the previous results, the inhibition of CTSB did not modify the As_2_O_3_-induced lipid accumulation (Fig. 4e). These results indicated that pyroptosis induced by As_2_O_3_ was alleviated by the CTSB inhibitors.

### Increased intracellular autophagy levels are associated with an increase in cytoplasmic CTSB, activation of NLRP3 inflammasome, and pyroptosis induced by As_2_O_3_

**Fig. 5:**
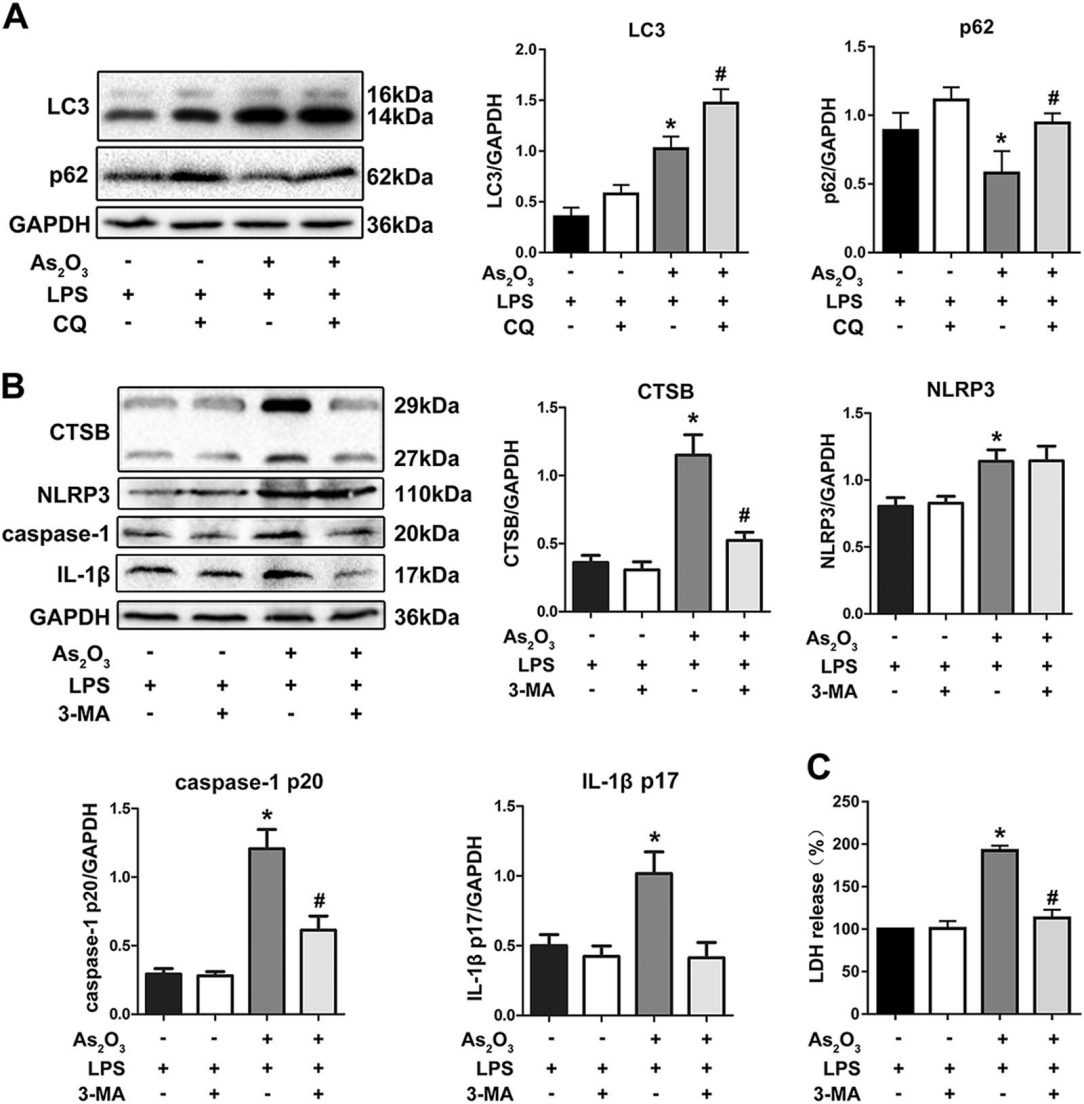
Increased intracellular autophagy levels associated with the activation of the NLRP3 inflammasome and the release of LDH induced by As_2_O_3_. **a** Autophagic flux analysis. **b** The effect of 3-MA on the expression of CTSB, caspase-1 activation, and production of IL-1β in As_2_O_3_-treated HepG2 cells. **b**–**e** Densitometric analyses of CTSB, NLRP3, caspase-1 p20, and IL-1β. **c** Effects of As_2_O_3_ and 3-MA on LDH release rate in HepG2 cells. Values are mean ± SD, and *n* = 3. *P < 0.05 compared with the control group, ^#^*P* < 0.05 compared with the As_2_O_3_ group

The increase in cytosolic CTSB levels is associated with lysosomal degradation, and our results show an increase in autophagic flux in hepatocytes. To further confirm the change of autophagic flux, we used the autophagy inhibitor chloroquine (CQ) in HepG2 cells. Upregulation of LC3 by As_2_O_3_ was potentiated by CQ, and the downregulation of p62 was reversed by CQ since CQ inhibited the fusion of autophagosome and autolysosome (Fig. 5a).

**Fig. 6:**
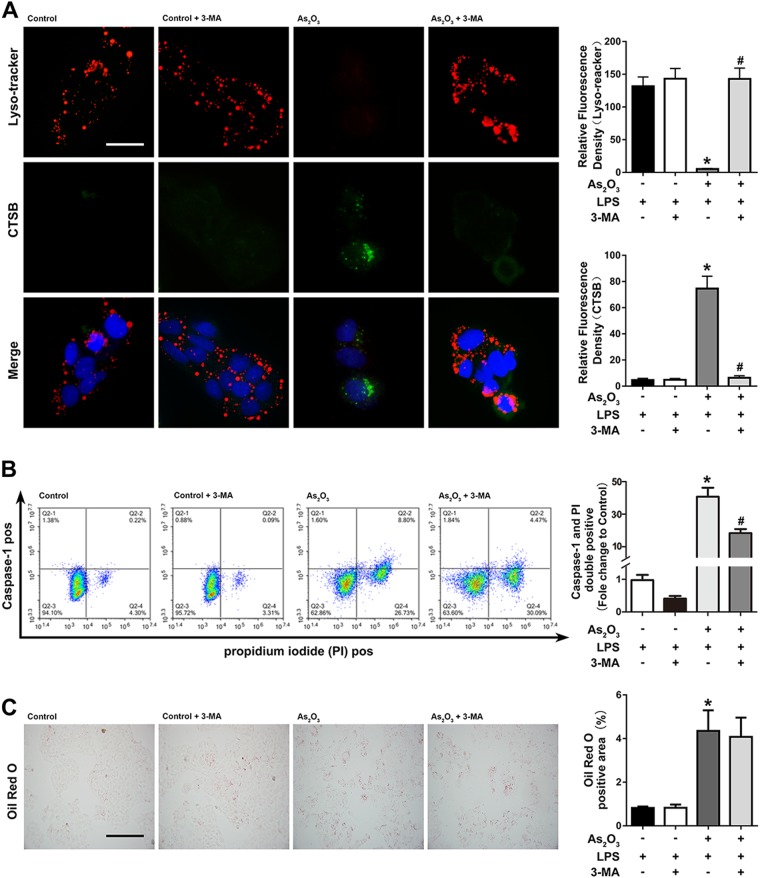
Effects of 3-MA on CTSB levels, pyroptosis, and lipid accumulation. **a** Detection of cytosolic and lysosomal CTSB levels after As_2_O_3_ and CA-074 Me Treatments. **b** Effects of 3-MA on As_2_O_3_-induced double-positivity for PI and activated caspase-1 staining. **c** Effects of 3-MA on the As_2_O_3_-induced accumulation of lipids. Values are mean ± SD, and *n* = 3. *P < 0.05 compared with the control group, ^#^*P* < 0.05 compared with the As_2_O_3_ group; Scale bar = 50 μm

To demonstrate the link between autophagy and the activation of the NLRP3 inflammasome, we pretreated with HepG2 cells with the autophagy inhibitor 3-MA. The expression of cytosolic CTSB, caspase-1 p20, IL-1β, and release of LDH were upregulated by As_2_O_3_ and downregulated by 3-MA (Fig. 5b, c). The increased cytosolic CTSB levels were also reversed by 3-MA (Fig. 6a). Moreover, As_2_O_3_-induced pyroptotic cell death was also reduced by 3-MA, which was indicated by decreased caspase-1 and PI double-positivity (Fig. 6b). Not surprisingly, the As_2_O_3_-induced accumulation of lipids was not relieved by 3-MA (Fig. 6c).

### Taurine decreases autophagy, NLRP3 inflammasome activation, and pyroptotic cell death caused by As_2_O_3_

**Fig. 7:**
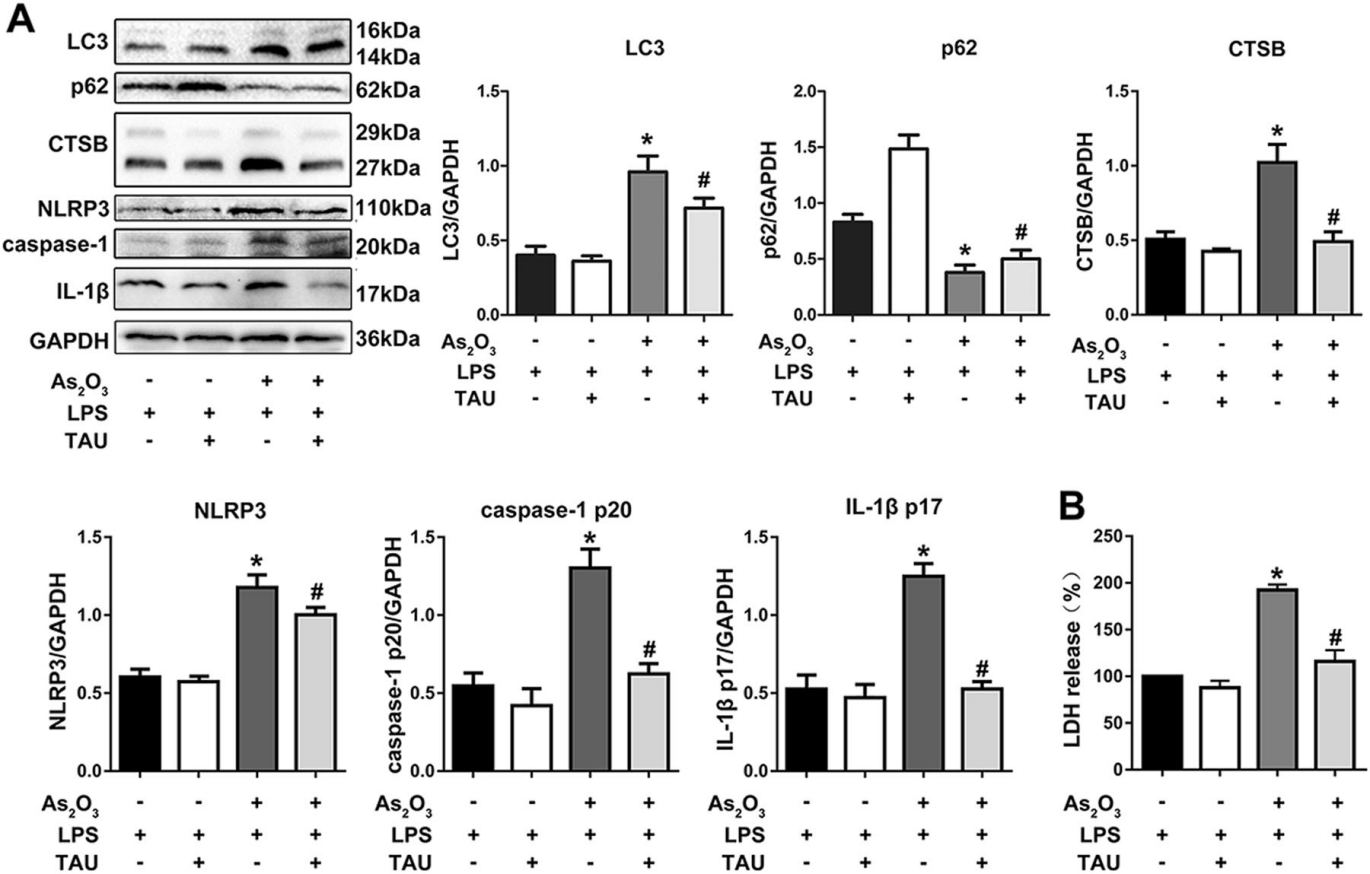
Taurine inhibited the elevated levels of autophagy, NLRP3 inflammasome activation, and the release of LDH caused by As_2_O_3_. **a** The effect of Tau on the expression of LC3, p62, CTSB, NLRP3, caspase-1 p20, and IL-1β p17 in As_2_O_3_-treated HepG2 cells, and densitometric analyses of LC3, p62, CTSB, NLRP3, caspase-1 p20, and IL-1β. **b** Effects of As_2_O_3_ and Tau on LDH release rate in HepG2 cells. Values are mean ± SD, and *n* = 3. *P < 0.05 compared with the control group, ^#^*P* < 0.05 compared with the As_2_O_3_ group

**Fig. 8:**
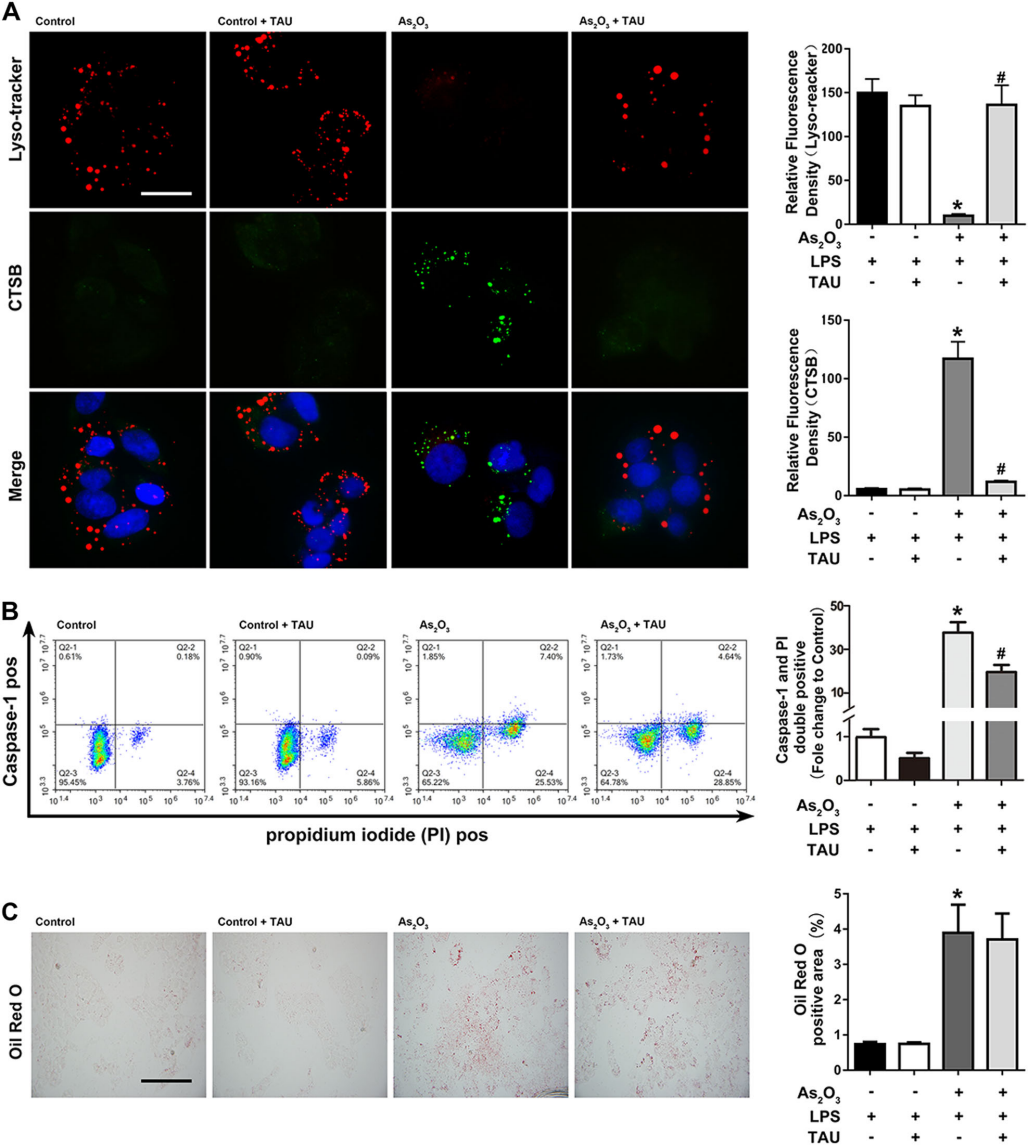
Effects of Tau on CTSB levels, pyroptosis, and lipid accumulation. **a** Detection of cytosolic lysosomal and CTSB Levels after As_2_O_3_ and Tau Treatments. **b** Effects of Tau on As_2_O_3_-induced double-positivity for PI and activated caspase-1 staining. **c** Effects of Tau on the As_2_O_3_-induced accumulation of lipids. Values are mean ± SD, and *n* = 3. *P < 0.05 compared with the control group, ^#^*P* < 0.05 compared with the As_2_O_3_ group; Scale bar = 50 μm

In mouse livers, we showed that the As_2_O_3_-induced inflammation was attenuated by pretreatment with Tau. Thus, we pretreated HepG2 cells with Tau to identify the potential molecular mechanism underlying this process. Autophagy, CTSB levels, NLRP3 inflammasome activation, IL-1β expression, and the release of LDH were increased after treatment with As_2_O_3_ but were all decreased by treatment with Tau (Fig. 7a, b). Simultaneously, the increased cytosolic CTSB levels and the pyroptotic cell death induced by As_2_O_3_ were reversed by Tau (Fig. 8a, b). Similar to the previous results, Tau did not alleviate the accumulation of lipid caused by As_2_O_3_ in vivo, and we observed the same phenomenon in HepG2 cells (Fig. 8c).

## Discussion

The present study demonstrated that As_2_O_3_ upregulated the level of autophagy and triggered NLRP3 inflammasome activation, leading to pyroptotic cell death; and these effects could be reversed by taurine. Additionally, we found that accumulation of lipid and dysregulation of lipid-associated genes caused by As_2_O_3_ was not attenuated by taurine. Furthermore, we demonstrated that As_2_O_3_-induced pyroptotic cell death depended upon the CTSB-mediated activation of NLRP3 inflammasome. In addition, As_2_O_3_-induced autophagy was implicated in the arsenic-induced release of CTSB, subsequent NLRP3 inflammasome activation, and pyroptotic cell death. We have shown that taurine inhibits arsenic-induced inflammation and pyroptosis via the autophagic-CTSB-inflammasomal pathway.

It is worth mentioning that, contrary to our findings, Lau. et al.^37^ found that low dose of As_2_O_3_ inhibits autophagy levels. This phenomenon may be related to the different dosage of As_2_O_3_. Many poisons have different effects at different dose levels, and As_2_O_3_ is no exception.

It has been reported that the activation of NLRP3 inflammasome is involved in NASH-associated liver cell pyroptosis, inflammation, and fibrosis^21,38^. The NLRP3 inflammasome acts as a novel PRR and can be activated by exogenous PAMPs (pathogen-associated molecular patterns) and endogenous DAMPs (damage-associated molecular patterns) released by cellular stress^39,40^. Pyroptosis is associated with cell swelling, the release of pro-inflammatory intracellular contents, and pore formation in the cell membrane, thereby obtaining the ability to internalize propidium iodide (PI) dye^41,42^. Using a novel flow cytometry method, we found a marked increase in the number of As_2_O_3_-treated HepG2 cells double-positive for active caspase-1 and PI, which was reversed using the NLRP3 inhibitor MCC950. These results indicate that As_2_O_3_ causes liver inflammation via activation of the NLRP3 inflammasome and pyroptotic cell death.

During the development of NASH disease, saturated fatty acids can induce activation of the NLRP3-ASC inflammasome, activate caspase-1, make IL-1β and mature IL-18, and autophagosomes and mitochondrial active oxygen species are also involved^43,44^. Moreover, in autophagy, cytoplasmic LC3 protein is processed and recruited to the autophagosomal membranes. The autophagosome then fuses with the lysosome, which results in the breakdown of the autophagosome and its contents. The ubiquitin-associated protein p62, which binds to LC3, is an important marker for the induction of autophagy, clearance of protein aggregates, and the inhibition of autophagy^45–47^. In our previous studies, we reported that NaAsO_2_ or As_2_O_3_ induced excessive autophagy and caused autophagic cell death in INS-1 cells or HepG2 cells^48,49^. In this study, the similar results were observed. Treatment with arsenic elevated the levels of LC3, CTSB, and caspase-1 p20, and decreased the level of p62 both in vitro and in vivo, suggesting that the degradation of autophagolysosome leads to release of CTSB into the cytosol and activation of the NLRP3 inflammasome. Additionally, as shown earlier and confirmed in our study, release of lysosomal contents and CTSB into the cytosol mediates the activation of NLRP3 inflammasome^50^. A decreased level of active caspase-1 and a decreased amount of pyroptotic cell death after treatment with the CTSB inhibitor were observed in As_2_O_3_-treated HepG2 cells. Furthermore, the increased level of CTSB and subsequent activation of NLRP3 inflammasome and pyroptotic cell death induced by As_2_O_3_ were reversed by the autophagy inhibitor 3-MA. These results demonstrate that CTSB contributes to the As_2_O_3_-induced activation of NLRP3 inflammasome, which was autophagy-dependent.

In contrast to the positive regulation of autophagy by the inflammasome in our research, a recent study observed enhanced maturation and secretion of IL-1β and IL-18 in macrophages and monocytes isolated from mice genetically deficient in Beclin 1 and LC3B^51^. Cytokine activation in response to LPS and ATP in wild-type macrophages, as well as amplification observed in LC3B or Beclin 1 deficient macrophages, required the NLRP3 inflammasome pathway^51,52^. When the autophagy regulatory genes atg16L1 or atg7 are knocked out, or autophagy inhibitors are used, LPS-dependent inflammasomes are activated, indicating that autophagy can regulate inflammatory body activation and limit generation of inflammatory IL-1β and IL-18^53^. However, similar to our results, autophagy induction by starvation enhances caspase-1 activation and secretion of IL-1β and IL-18^54^. Inflammasome-mediated IL-1β secretion utilizes the autophagy-based unconventional secretion pathway^54^. It is possible that a distinct type of autophagy induction might differentially regulate the inflammasome pathway^55^. Taken together, these studies suggest an important role for autophagy proteins in proinflammatory responses, which warrant further investigation in models of inflammatory disease^55^.

Interestingly, in our study, inhibition of autophagy, CTSB, NLRP3 inflammasomes, or application of taurine did not improve As_2_O_3_-induced lipid accumulation. Additionally, taurine did not improve the arsenic-induced dysregulation of lipid-related genes in mouse livers, but autophagy and NLRP3-inflammasome activation were both inhibited. These results indicate that taurine relieves NASH caused by chronic arsenic exposure by inhibiting autophagic-inflammasomal pathways rather than improving lipid accumulation. Our previous studies suggested that taurine inhibited arsenic-induced excessive autophagy by the PPARγ pathway. Additionally, Ralston et al. used saturated palmitic acid to simulate saturated fatty acids and showed that saturated fatty acids could activate NLRP3 inflammasomes. However, they did not define its explicit molecular mechanisms^56^. Therefore, in the future studies, we will further explore the specific targets of taurine inhibition of autophagy and cell death, as well as the specific mechanism and role of lipid droplet formation induced by As_2_O_3_ in inflammasome activation and pyroptosis.

**Fig. 9:**
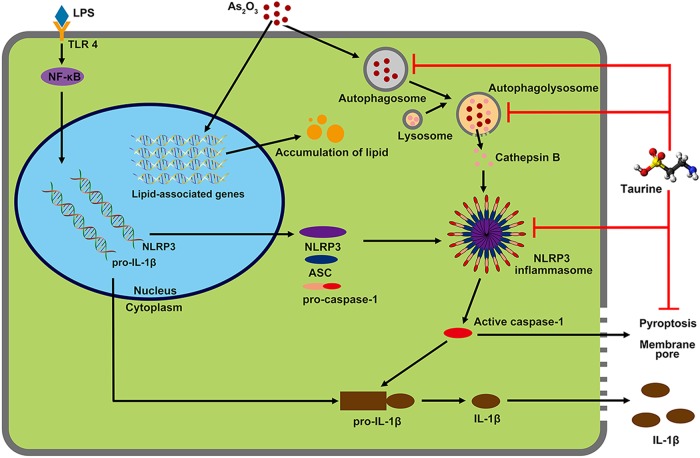
Synoptic model of As_2_O_3_-induced hepatotoxicity. Chronic exposure to As_2_O_3_ induces dysregulation of lipid-associated genes and accumulation of lipids. Moreover, As_2_O_3_ induces elevated autophagy levels, autophagosomal degrading, and increased cytoplasmic lysosomal-content CTSB in hepatocytes. Increased CTSB causes NLRP3 inflammasome activation, which leads to IL-1β maturation and pyroptosis. The accumulated lipids and secreted cytokines terminally result in NASH

In conclusion, this study indicates that the NLRP3 inflammasome is involved in arsenic-induced NASH, and the molecular mechanism of NLRP3 inflammasome activation underlies the autophagy and degradation of autophagolysosome caused by arsenic exposure. We also show that taurine improves arsenic-induced NASH by inhibiting autophagic-inflammasome pathways rather than reducing lipid accumulation (Fig. 9). Therefore, this study provides a novel insight into arsenic-induced hepatotoxicity and the potential molecular mechanism by which arsenic causes NASH. This knowledge may be utilized to improve therapeutic strategies for arsenicosis.

## Materials and methods

### Animals

Six-week-old C57BL/6J mice were purchased from the Institute of Genome Engineered Animal Models for Human Disease of Dalian Medical University. To investigate the effect of As_2_O_3_ on the liver and the protective capability of taurine, the mice were exposured to As_2_O_3_ (Sigma-Aldrich, 132753-3) at doses of 0, 1, 2, and 4 mg/L in drinking water for 12 weeks, and the taurine group was given 4 mg/L As_2_O_3_ in drinking water and 250 mg/kg taurine by gavage daily. The control group was given distilled water (*n* = 6 for each group). At the end of the experimental period, the animals were euthanized, and the livers were collected for the following experiments.

### Cell culture

Human hepatoma cell line HepG2 was purchased from the American Type Culture Collection (ATCC). HepG2 cells were cultured in MEM/EBSS (Gibco, 1853128) medium supplemented with 10% fetal bovine serum (Biological Industries, 1707254) and antibiotics (Hyclone, HYC-SV30010) under a humidified atmosphere with 5% CO_2_ at 37 °C. 3.96 mg of As_2_O_3_ was dissolved in 1 ml phosphate buffered saline (PBS) to prepare a stock solution of 20 μM, and HepG2 cells were treated with 0 or 4 μM As_2_O_3_ (Sigma-Aldrich, 132753-3) for 48 h.

### Pretreatment of cells

Before As_2_O_3_-treatment, cells were pre-treated with 0.5 mM of the autophagy inhibitor chloroquine (Sigma-Aldrich, C6628), 3-MA (Santa Cruz, sc-205596), 5 μM CTSB inhibitor CA-074 Me (AdooQ, A13256), NLRP3 inhibitor MCC950 (MCE, HY-12815A), or 150 μM taurine, and 1 μg/ml lipopolysaccharides (LPS, Sigma-Aldrich, L4391) for 4 h.

### Cell viability assay

The cytotoxicity of As_2_O_3_ was detected by MTT assay. HepG2 cells (5 × 10^4^/ml) were seeded in 96-well plates and treated with 0, 2, 4, 8, 16, or 32 μM As_2_O_3_ for 24 or 48 h. After treatment, 0.5 mg/ml MTT (Solarbio, M8180) was added, and the cells were incubated for 4 h at 37 °C. The supernatant was discarded, and then 100 μl DMSO was added into each well for 30 min. The plate was gently agitated until the blue formazan crystals were fully dissolved. The cell viability was measured at 570 nm using a Bio-Rad Microplate Reader, and the cell viability (%) was calculated using the following equation: (A570 of treated sample/A570 of control) × 100.

#### LDH release assay

After the cells were treated with As_2_O_3_ 48 h, the culture supernatants were collected. LDH levels in the supernatants were determined using an LDH Cytotoxicity Assay Kit (Beyotime, C0016) according to the manufacturer’s protocol. The absorbance was read at 490 nm with a microplate reader (Thermo Fisher Scientific).

### Histopathological examination

Liver tissues from C57BL/6 mice were fixed in 10% neutral buffered formalin, dehydrated in graded volumes of ethanol alcohols, embedded in paraffin, sectioned at 4 µm thickness, and stained with hematoxylin and eosin (H&E) for microscopic examination. Liver slides were observed under the light microscope (Olympus BX63, 40 × 10) and examined in a blinded fashion.

### Oil Red O staining

5 μm thick frozen sections of mouse livers or treated HepG2 cells were washed twice with PBS and fixed in 4% paraformaldehyde at room temperature for 40 min. After two washes in 60% isopropyl alcohol, the slices or cells were stained with an Oil Red O stain kit (KeyGEN, KGA329) according to the manufacturer’s protocol. After removing the Oil Red O solution, the slices or cells were washed with distilled water five times and observed under a microscope (Olympus BX63, 40 × 10). The staining intensity of Oil Red O was quantified with ImageJ software.

### Triglyceride content detection

TG levels in liver were analyzed using a Triglycerides Assay Kit (Nanjing Jiancheng Bioengineering Institute, A110-1). Briefly, small portions of liver tissue (50 mg) were collected and homogenized in 100% ethanol (450 mL). After centrifugation, the supernatant was collected for analysis based on the glycerol lipase oxidase (GPO-PAP) method. Samples were reacted with the mixture from the kit and were incubated at 37 °C for 10 min, and absorbance at 510 nm was read with a microplate reader (Thermo Fisher Scientific).

### Transmission electron microscopy

The autophagosomes of hepatocytes were observed with transmission electron microscopy as described previously^49^. Parts of liver were fixed with 1.25% glutaraldehyde for 1 day, and then post-fixed in 1% osmium tetroxide for 1 h. Dehydration was done in a concentration gradient of ethanol followed by propylene oxide. While incubated in 70% ethanol, the pellet was stained en bloc with 1% uranyl acetate. Finally, the pellet was embedded in Epon resin. Ultrathin sections were post-stained with uranyl acetate and Reynold’s lead citrate routinely. Electron micrographs were taken with a JEM 1400 transmission electron microscope at 80 kV.

### NAFLD activity score (NAS)

Mouse livers were scored under blinded conditions utilizing the NAFLD activity score (NAS). This score is defined as the unweighted sum of the scores for steatosis (0–3), lobular inflammation (0–3), and ballooning (0–2). Scores of 0–2 are not NASH, 3–4 are considered borderline, and 5–8 are considered NASH^8^.

#### Pyroptosis detection

##### Liver frozen slide

The pyroptotic cells were detected with FLICA® 660-YVAD-FMK (ImmunoChemistry Technologies, NRM-KF17362) by immunofluorescence, according to the manufacturer’s protocol. The surfaces of the tissues were covered in tissue section staining solution (TSSS) and incubated for 30–60 min while protected from light. The slides were then washed with 1× Cellular Wash Buffer twice for 5 min each. The slides were then set in an incubation dish containing 1× Cellular Wash Buffer. Nuclei were stained with DAPI (ROCHE, 28718-90-3) for 20 min at room temperature and protected from light. The slides were then imaged with a fluorescence microscope (Olympus BX63, 40 × 10).

##### HepG2 cells

The pyroptotic cells were detected by flow cytometry (FCM). After 48 h of exposure to As_2_O_3_, HepG2 cells were harvested in 1.5 mL centrifuge tubes for following experiments: active caspase-1 was measured in HepG2 cells with FLICA 660-YVAD-FMK, and membrane pore formation was measured by staining with propidium iodide (PI, KeyGEN, KGA214) according to the manufacturer’s instructions. After staining, the cells were analyzed using an ACEA NovoCyte flow cytometer (NovoCyte 2040 R), and pyroptosis was defined as double positive for caspase-1 and PI.

### Western blot

Proteins were extracted from the HepG2 cells or from part of the mouse liver, lysed with cell lysis buffer (Merck Millipore, 92590) containing 1 mM phenylmethylsulfonyl fluoride (PMSF, KeyGEN, KGP610), 1 mM protease inhibitor (KeyGEN, KGP603), and 1 mM phosphatase inhibitors (KeyGEN, KGP602) and then quantified with the BCA Protein Assay kit (Thermo, MK164230). Total proteins were separated by sodium dodecyl sulfate-polyacrylamide gel electrophoresis (SDS-PAGE) and blotted to polyvinylidene fluoride (PVDF) membranes (Merck Millipore, ISEQ00010) via a wet electrophoretic transfer method. Membranes were blocked for 1 h with 10% powdered skimmed milk by gentle shaking at 37 °C in a water bath shaker and then incubated with primary antibodies against LC3B (Abcam, ab192890, 1:1000), LAMP1 (Abcam, ab24170 1:800), p62 (Proteintach, 18420-1-AP, 1:1000), CTSB (CST, #31718, 1:1000), NLRP3 (CST, #15101, 1:800), ASC (CST, #67824, 1:1000), caspase-1 (Abcam, ab1872, 1:1000), GAPDH (Proteintech, 10494-1-AP, 1:1000), IL-1β (Abcam, ab9787, 1:1000), IL-6 (Abcam, ab208113, 1:1000), IL-10 (Abcam, ab189392, 1:1000), TNF-α (Abcam, ab66579, 1:1000), or MPO (Abcam, ab208670, 1:1000) at 4 °C overnight. After washing with PBS, the membranes were incubated with HRP-conjugated secondary antibody for 1.5 h on a shaker at room temperature or at 4 °C overnight. Protein expression levels were detected using an ECL kit (Beyotime, P0018), imaged on a Bio-Rad ChemiDoc TMMP system, and analyzed by ImageJ software.

### Quantitative real-time PCR

Total RNA from mouse livers was isolated using RNAiso Plus (Takara). Samples were prepared for RT-PCR using the PrimeScript ™ RT reagent Kit (Takara, RR036A) and SYBR Premix Ex TaqTM (Takara, RR820A), and RT-PCR was performed using a Rotor-Gene Q instrument (Qiagen). The relative expression of the target genes was calculated by the ΔΔCt method and each sample was done in triplicate. The sequences of primers were as follows:

**Table:** 

Gene name	Forward	Reverse
SREBP-1c	5′-CAGACTCACTGCTGCTGACA-3′	5′-GATGGTCCCTCCACTCACCA-3′
FAS	5′-GGCCCCTCTGTTAATTGGCT-3′	5′-GGATCTCAGGGTTGGGGTTG-3′
ACS	5′-ATCAGGCTGCTTATGGACGA-3′	5′-ATCCCACAGGCTGTTGTTTC-3′
SCD-1	5′-GTACCGCTGGCACATCAACT-3′	5′-AACTCAGAAGCCCAAAGCTCA-3′
PPARα	5′-TGCCTTCCCTGTGAACTGAC-3′	5′-TGGGGAGAGAGGACAGATGG-3′
ACC	5′-GCCTCAGGAGGATTTGCTGT-3′	5′-AGGATCTACCCAGGCCACAT-3′
HAD	5′-AAAACACCGATGACCAGCCA-3′	5′-TCTTCCTTAGACGCATCGCC-3′
CPT-1	5′-GGACTCCGCTCGCTCATT-3’	5′-GAGATCGATGCCATCAGGGG-3′
GAPDH	5′-ATCACTGCCACCCAGAAGAC-3′	5′-AGATCCACGACGGACACATT-3′

### Immunofluorescence staining

#### Liver frozen slide

Liver slices 5 μm thick were placed on IHC slides and fixed with 4% paraformaldehyde for 20 min. Non-specific binding was blocked with buffer containing 10% bovine serum albumin (BSA, Solarbio, A8020) for 1 h at room temperature. Liver slices were incubated overnight at 4 °C with anti-F4/80 (Abcam, ab6640), diluted at 1:200 in blocking buffer. After the slides were washed, they were incubated in the dark at room temperature for 30 min with Alexa Fluor 488 secondary antibodies (Proteintech, SA00006-2) diluted at 1:400 in blocking buffer. The slices were then washed with PBS and stained with DAPI (ROCHE, 28718-90-3) for 20 min at room temperature. The slides were imaged with a fluorescence microscope (40 × 10) and analyzed using Image-Pro Plus 7.0.

#### HepG2 cells

To observe the distribution of CTSB in the cytoplasm, we incubated slides with primary rabbit monoclonal antibodies against CTSB (CST, #31718, 1:200) after fixation and blocking, and lysosomes were labeled with Lysotracker Red (Beyotime). Alexa Fluor 488 secondary antibodies (Proteintech, SA00006-2) diluted at 1:400 in blocking buffer where then added in the dark. We analyzed the 4,6-diamidino-2-phenylindole (DAPI) counterstained slides under a fluorescence microscope (40 × 10).

### Statistical analysis

Data are expressed as means ± standard deviation (SD) from at least three independent experiments performed in triplicate. The data were analyzed using SPSS 13.0 (SPSS Inc., Chicago, IL, USA). Significance was determined using one-way analysis of variance (ANOVA) or t-test. *P* values were all two-sided, and a *P* value < 0.05 was considered as statistically significant.

## References

[1] Rodriguez-Lado L (2013). Groundwater arsenic contamination throughout China. Science.

[2] Chen CJ (2005). Biomarkers of exposure, effect, and susceptibility of arsenic-induced health hazards in Taiwan. Toxicol. Appl. Pharmacol..

[3] Singh AP, Goel RK, Kaur T (2011). Mechanisms pertaining to arsenic toxicity. Toxicol. Int..

[4] Santra A, Das Gupta J, De BK, Roy B, Guha Mazumder DN (1999). Hepatic manifestations in chronic arsenic toxicity. Indian J. Gastroenterol..

[5] Lu T (2001). Application of cDNA microarray to the study of arsenic-induced liver diseases in the population of Guizhou, China. Toxicol. Sci..

[6] Fry RC (2007). Activation of inflammation/NF-κB signaling in infants born to arsenic-exposed mothers. PLoS Genet..

[7] Das N (2012). Arsenic exposure through drinking water increases the risk of liver and cardiovascular diseases in the population of West Bengal, India. BMC Public. Health.

[8] Ditzel EJ, Nguyen T, Parker P, Camenisch TD (2016). Effects of arsenite exposure during fetal development on energy metabolism and susceptibility to diet-induced fatty liver disease in male mice. Environ. Health Perspect..

[9] Tan M (2011). Chronic subhepatotoxic exposure to arsenic enhances hepatic injury caused by high fat diet in mice. Toxicol. Appl. Pharmacol..

[10] Chalasani N (2012). The diagnosis and management of non‐alcoholic fatty liver disease: Practice Guideline by the American Association for the Study of Liver Diseases, American College of Gastroenterology, and the American Gastroenterological Association. Hepatology.

[11] Whalley S, Puvanachandra P, Desai A, Kennedy H (2007). Hepatology outpatient service provision in secondary care: a study of liver disease incidence and resource costs. Clin. Med..

[12] Fan JG (2011). Guidelines for the diagnosis and management of nonalcoholic fatty liver disease: update 2010. J. Dig. Dis..

[13] Bedogni G (2005). Prevalence of and risk factors for nonalcoholic fatty liver disease: the Dionysos nutrition and liver study. Hepatology.

[14] Amarapurkar DN (2007). How common is non‐alcoholic fatty liver disease in the Asia–Pacific region and are there local differences?. J. Gastroenterol. Hepatol..

[15] Byrne CD, Targher G (2015). NAFLD: a multisystem disease. J. Hepatol..

[16] Ekstedt M (2006). Long-term follow-up of patients with NAFLD and elevated liver enzymes. Hepatol. (Baltim., Md).

[17] Ganz M, Szabo G (2013). Immune and inflammatory pathways in NASH. Hepatol. Int..

[18] Sutti S, Bruzzì S, Albano E (2016). The role of immune mechanisms in alcoholic and nonalcoholic steatohepatitis: a 2015update. Expert Rev. Gastroenterol. & Hepatol..

[19] Schroder K, Tschopp J (2010). The inflammasomes. Cell.

[20] Lu A (2014). Unified polymerization mechanism for the assembly of ASC-dependent inflammasomes. Cell.

[21] Wree A (2014). NLRP3 inflammasome activation results in hepatocyte pyroptosis, liver inflammation, and fibrosis in mice. Hepatology.

[22] Lamkanfi M, Dixit VM (2014). Mechanisms and functions of inflammasomes. Immunol. Rev..

[23] Man Si Ming, Karki Rajendra, Kanneganti Thirumala-Devi (2017). Molecular mechanisms and functions of pyroptosis, inflammatory caspases and inflammasomes in infectious diseases. Immunological Reviews.

[24] de Roos B (2009). Attenuation of inflammation and cellular stress‐related pathways maintains insulin sensitivity in obese type I interleukin‐1 receptor knockout mice on a high‐fat diet. Proteomics.

[25] Abderrazak A (2015). NLRP3 inflammasome: from a danger signal sensor to a regulatory node of oxidative stress and inflammatory diseases. Redox Biol..

[26] Ozaki E, Campbell M, Doyle SL (2015). Targeting the NLRP3 inflammasome in chronic inflammatory diseases: current perspectives. J. Inflamm. Res..

[27] Strowig T, Henao-Mejia J, Elinav E, Flavell R (2012). Inflammasomes in health and disease. Nature.

[28] Lu Y (2016). CdSe/ZnS quantum dots induce hepatocyte pyroptosis and liver inflammation via NLRP3 inflammasome activation. Biomaterials.

[29] Blondelle J, Lange S, Greenberg BH, Cowling RT (2015). Cathepsins in heart disease-chewing on the heartache?. Am. J. Physiol. Heart Circ. Physiol..

[30] Li S (2013). Cathepsin B contributes to autophagy-related 7 (Atg7)-induced nod-like receptor 3 (NLRP3)-dependent proinflammatory response and aggravates lipotoxicity in rat insulinoma cell line. J. Biol. Chem..

[31] Geronimo-Olvera C, Montiel T, Rincon-Heredia R, Castro-Obregon S, Massieu L (2017). Autophagy fails to prevent glucose deprivation/glucose reintroduction-induced neuronal death due to calpain-mediated lysosomal dysfunction in cortical neurons. Cell Death Dis..

[32] Wang D (2017). The role of NLRP3-CASP1 in inflammasome-mediated neuroinflammation and autophagy dysfunction in manganese-induced, hippocampal-dependent impairment of learning and memory ability. Autophagy.

[33] Jiang N (2015). An integrated metabonomic and proteomic study on Kidney-Yin Deficiency Syndrome patients with diabetes mellitus in China. Acta Pharmacol. Sin..

[34] Ito T, Schaffer SW, Azuma J (2012). The potential usefulness of taurine on diabetes mellitus and its complications. Amino Acids.

[35] Song M, Salam NK, Roufogalis BD, Huang THW (2011). Lycium barbarum (Goji Berry) extracts and its taurine component inhibit PPAR-γ-dependent gene transcription in human retinal pigment epithelial cells: possible implications for diabetic retinopathy treatment. Biochem. Pharmacol..

[36] Murakami S., Yamori Y. Taurine and longevity–preventive effect of taurine on metabolic syndrome. *Bioactive Food as Dietary Interventions for the Aging Population*, 159–171 (2013).

[37] Lau A (2013). Arsenic inhibits autophagic flux, activating the Nrf2-Keap1 pathway in a p62-dependent manner. Mol. Cell. Biol..

[38] Wree A (2014). NLRP3 inflammasome activation is required for fibrosis development in NAFLD. J. Mol. Med..

[39] Arrese M, Cabrera D, Kalergis AM, Feldstein AE (2016). Innate immunity and inflammation in NAFLD/NASH. Dig. Dis. Sci..

[40] Abderrazak A (2015). NLRP3 inflammasome: from a danger signal sensor to a regulatory node of oxidative stress and inflammatory diseases. Redox Biol..

[41] Shao W, Yeretssian G, Doiron K, Hussain SN, Saleh M (2007). The caspase-1 digestome identifies the glycolysis pathway as a target during infection and septic shock. J. Biol. Chem..

[42] Brennan MA, Cookson BT (2000). Salmonella induces macrophage death by caspase‐1‐dependent necrosis. Mol. Microbiol..

[43] Csak T (2011). Fatty acid and endotoxin activate inflammasomes in mouse hepatocytes that release danger signals to stimulate immune cells. Hepatology.

[44] Wen H (2011). Fatty acid-induced NLRP3-ASC inflammasome activation interferes with insulin signaling. Nat. Immunol..

[45] Jiang P, Mizushima N (2015). LC3-and p62-based biochemical methods for the analysis of autophagy progression in mammalian cells. Methods.

[46] Schläfli AM (2016). Prognostic value of the autophagy markers LC3 and p62/SQSTM1 in early-stage non-small cell lung cancer. Oncotarget.

[47] Pankiv S (2007). p62/SQSTM1 binds directly to Atg8/LC3 to facilitate degradation of ubiquitinated protein aggregates by autophagy. J. Biol. Chem..

[48] Zhu XX (2014). Sodium arsenite induces ROS-dependent autophagic cell death in pancreatic β-cells. Food Chem. Toxicol..

[49] Bai J. et al. Taurine protects against As_2_O_3_-induced autophagy in livers of rat offsprings through PPARγ pathway. *Sci. Rep.*, **6** 1–14 (2016). 10.1038/srep27733 PMC4904213 27291853

[50] Li S (2013). Cathepsin B contributes to autophagy-related 7 (Atg7)-induced nod-like receptor 3 (NLRP3)-dependent proinflammatory response and aggravates lipotoxicity in rat insulinoma cell line. J. Biol. Chem..

[51] Nakahira K (2011). Autophagy proteins regulate innate immune responses by inhibiting the release of mitochondrial DNA mediated by the NALP3 inflammasome. Nat. Immunol..

[52] Zhou R, Yazdi AS, Menu P, Tschopp J (2011). A role for mitochondria in NLRP3 inflammasome activation. Nature.

[53] Puleston DJ, Simon AK (2014). Autophagy in the immune system. Immunology.

[54] Dupont N (2011). Autophagy‐based unconventional secretory pathway for extracellular delivery of IL-1β. EMBO J..

[55] Ryter SW, Mizumura K, Choi AMK (2014). The impact of autophagy on cell death modalities. Int. J. Cell Biol..

[56] Ralston JC, Lyons CL, Kennedy EB, Kirwan AM, Roche HM (2017). Fatty acids and NLRP3 inflammasome–mediated inflammation in metabolic tissues. Annu. Rev. Nutr..

